# Why I Should Keep Accurate Records of Business Transactions

**Published:** 1919-12

**Authors:** 


### WHY I SHOULD KEEP ACCURATE RECORDS OF BUSINESS TRANSACTIONS

Because I am a "two-handed earner." My income is largely dependent on the labors of my own hands, though I am assisted by others.

Because there are only a few years between now and the age of 55, after which I probably can not earn more than a living. Whatever I do in accumulating a competency must be done now.

Because I have probably not more than 1,000 income hours for each of the years of my working period. If each of these hours does not produce a profit, there is no financial hope for me in old age.

Because my office expenses are not incidental, accidental or uncertain. My recognition of them may be of these, but the expenses are constant, certain and fixed. They work 2,000 hours per year.

Because my fees can not be fair to patients and myself unless I know my office expense, my proper remuneration, my operating cost per income hour and the average time required for each operation.

Because the tradition-determined fees or the competition-determined fees are not suited to modern technic and requirements and are evidently unprofitable for many operations, such as root canal treatment, prophylaxis, consultations and plate-making.

Because if my conduct is so unbusinesslike that I have to work overtime to make a living, I can not do any professional reading or make professional advancement. By standing still professionally I defraud my best patients and lower the standing of my profession.

Because I should like to put my service on a basis which will satisfy my professional conscience. That is, I should like to learn how to sell to patients the service that is best for them at a price that is fair to me. The first step in such selling is to learn what the price should be, and be able to justify that price against low-priced competition.

Because if I could know in the beginning of the year how much net income will be available for personal expenditure, as an employer knows, my family expenses could be adjusted and financial worry avoided.

Because if I can master a system of records that will give all this information, and can find out what I am doing and what I should do, it is unfair to my patients, to my family and to myself to decline or defer to begin learning because of the trouble it requires.—*Digest.*

